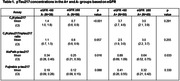# Effect of kidney function on plasma pTau217 concentrations using different pTau217 assays

**DOI:** 10.1002/alz.088364

**Published:** 2025-01-09

**Authors:** Alicia Algeciras‐Schimnich

**Affiliations:** ^1^ Mayo Clinic, Rochester, MN USA

## Abstract

**Background:**

Chronic kidney disease (CKD) has been associated with increased plasma phosphorylated Tau217 (pTau217) concentrations, potentially confounding its utility in the evaluation of Alzheimer's disease (AD). We assessed the association of estimated glomerular filtration rate (eGFR) with plasma pTau217 concentrations measured by various assays.

**Method:**

We included 195 participants from the Mayo Clinic Study of Aging or the Alzheimer’s Disease Research Center with diagnoses of cognitively unimpaired (n=114), mild cognitive impairment (n=70), and AD dementia (n=11). Amyloid‐PET was performed with Pittsburgh ^11^C‐Compound B; abnormal amyloid (A+) was defined as SUVR ≥1.52 (Centiloid ≥25). eGFR was determined based on serum creatinine concentrations. pTau217 was quantified using ALZpath Simoa pTau217 immunoassay, Fujirebio Lumipulse G pTau217(N‐Terminal) immunoassay, and a pTau217 mass spectrometry (MS) assay (C_2_N Diagnostics). Additionally, a pTau217 ratio (pTau217 to non‐phosphorylated [npTau217]) was calculated using the MS assay. Linear regression was used to assess associations of continuous eGFR with plasma pTau217 and amyloid PET. Differences in mean plasma concentrations between eGFR groups by amyloid PET status were evaluated with one‐way ANOVA tests and associated confidence intervals for each sample mean.

**Result:**

Median pTau217 concentrations in the A+ and A‐ groups were higher in individuals with eGFR <60 mL/min/1.73 m^2^ compared to those with eGFR ≥60 in all assays except for the A+ group when using the pTau217/npTau217 ratio. A 10‐unit worsening of eGFR was associated with higher pTau217 mean concentrations as follows: 24% (95% CI, 13‐36%) for C_2_N pTau217, 16% (9‐24%) for ALZpath pTau217, and 12% (5‐20%) for Lumipulse pTau217. eGFR was not significantly associated with the pTau217/npTau217 ratio or amyloid‐PET.

**Conclusion:**

CKD was associated with increased pTau217 concentrations when measuring pTau217 but not when using the pTau217 ratio. Additional research is needed to better understand the confounding effects of CKD in the clinical interpretation of plasma pTau217 assays.